# Development and initial evaluation of a novel simulation model for comprehensive brain tumor surgery training

**DOI:** 10.1007/s00701-020-04359-w

**Published:** 2020-05-08

**Authors:** Anne Sophie Grosch, Timo Schröder, Torsten Schröder, Julia Onken, Thomas Picht

**Affiliations:** 1 Department of Neurosurgery, Charité, Universitätsmedizin Berlin, Charitéplatz 1, 10117 Berlin, Germany; 2 Berliner Simulations & Trainingszentrum (BeST), Berlin Simulation Centre, Charité, Universitätsmedizin Berlin, Berlin, Germany; 3 Cluster of Excellence, Matters of Activity, Image Space Material, Humboldt University, Berlin, Germany

**Keywords:** Tumor model, Tumor resection, Training, Simulation, Neurosurgery, Surgical microscope

## Abstract

**Background:**

Increasing technico-manual complexity of procedures and time constraints necessitates effective neurosurgical training. For this purpose, both screen- and model-based simulations are under investigation. Approaches including 3D printed brains, gelatin composite models, and virtual environments have already been published. However, quality of brain surgery simulation is limited due to discrepancies in visual and haptic experience. Similarly, virtual training scenarios are still lacking sufficient real-world resemblance. In this study, we introduce a novel simulator for realistic neurosurgical training that combines real brain tissue with 3D printing and augmented reality.

**Methods:**

Based on a human CT scan, a skull base and skullcap were 3D printed and equipped with an artificial dura mater. The cerebral hemispheres of a calf’s brain were placed in the convexity of the skullcap and tumor masses composed of aspic, water, and fluorescein were injected in the brain. The skullcap and skull base were placed on each other, glued together, and filled up with an aspic water solution for brain fixation. Then, four surgical scenarios were performed in the operating room as follows: (1) simple tumor resection, (2) complex tumor resection, (3) navigated biopsy via burr hole trepanation, and (4) retrosigmoidal craniotomy. Neuronavigation, augmented reality, fluorescence, and ocular—as well as screen-based (exoscopic)—surgery were available for the simulator training. A total of 29 participants performed at least one training scenario of the simulator and completed a 5-item Likert-like questionnaire as well as qualitative interviews. The questionnaire assessed the realism of the tumor model, skull, and brain tissue as well as the capability for training purposes.

**Results:**

Visual and sensory realism of the skull and brain tissue were rated,”very good,” while the sensory and visual realism of the tumor model were rated “good.” Both overall satisfaction with the model and eligibility of the microscope and neurosurgical instruments for training purposes were rated with “very good.” However, small size of the calf’s brain, its limited shelf life, and the inability to simulate bleedings due to the lack of perfusion were significant drawbacks.

**Conclusion:**

The combination of 3D printing and real brain tissue provided surgical scenarios with very good real-life resemblance. This novel neurosurgical model features a versatile setup for surgical skill training and allows for efficient training of technological support like image and fluorescence guidance, exoscopic surgery, and robotic technology.

**Electronic supplementary material:**

The online version of this article (10.1007/s00701-020-04359-w) contains supplementary material, which is available to authorized users.

## Introduction

Previous studies showed that neurosurgical upbringing, especially brain tumor surgery, lacks high-quality training situations within or outside the operating theater. Training is still dominated by conventional mentor apprentice scenarios. Consequently, neurosurgical simulators gain in importance. Experiences with simulations using 3D prints, gelatin composites, virtual reality (VR), or cadaver trainings have already been reported (Table 1). Although VR trainings in particular are increasingly explored, they still do not provide scenarios capable of simulating the procedure of brain tumor resections as such. Here, training setups for comprehensive brain tumor resections distinctly benefit from the usage of real brain tissue [12]. Neurosurgical residence is characterized by enhanced diversification of procedures with increasingly sophisticated technological support. This requires expert knowledge and novel ways of human machine interaction. In parallel, requirements for quality control and standardization gain in importance. The consequence of the highly condensed and demanding neurosurgical working environment is an urgent need for training scenarios that effectively shorten the conventional learning curves of the “see one, do one, teach one” era [19]. Results of a recent survey demonstrated that European residents, and German residents in particular, are not satisfied with their theoretical and practical neurosurgical training and highlights the need for innovative training concepts [18]. On top of that, surgical simulation influences the translational outcome from theoretical knowledge to daily practice [16] and can provide motivational impact on the neurosurgical career [11].

**Table 1:** This table pictures an extract of current available neurosurgical training simulators that use different approaches for material composition and technology

Simulator	Operating principle	Surgical scenarios	Strengths	Limitations
NeuroTouch [10]	VR	Resection of a meningioma-like lesion	Able to differentiate participants by their training level, performance metrics were recorded automatically	Visual and sensory realism only “acceptable”
Plug-and-play lifelike ETV training model [20]	3D prints and casting/molding	Endoscopic third ventriculostomy (ETV) in pediatric hydrocephalus	Realistic human-like external features; pulsation of ventricular cavities, basilar artery, and flow of CSF; plug-and-play component allows for reuse	Expenditure of money and time, merely one pathological condition, extremely realistic external facial features were not superior to low-fidelity training model
MARTYN [8]	polyurethane resin (skull), gelatin composite base (brain), paraffin (CSF), latex (dura mater), silicone (temporalis muscle)	Frontal/temporal craniotomies; insertion of external ventricular drains (EVD) via burr holes; evacuation of extradural hematomas	Inexpensive, accessible, various pathologies possible, no tissue act restrictions	Inevitable minor variabilities, expenditure of time
Mixed reality simulation [5]	3D prints in combination with a virtual radiographic system or image guidance platform	Ventriculostomy; percutaneous stereotactic lesion procedure for trigeminal neuralgia; spinal instrumentation	Appropriate real-world visual and haptic feedback, scanning is possible	Does not include fluids or nerves
Agar agar tumor model [12]	Injecting a mixture of fluorescein and agar agar in a sheep’s brain	Corticotomy and successive complete dissection of a defined gyrus using a dissector, suction and ultrasound aspirator, neurosurgical tumor resection	Cheap, easily accessible, simple, realistic haptic feedback, fluorescent in 5-ALA	No training of craniotomies, neurosurgical approaches or identification of bony landmarks, no use in the OR due to sanitary regulations
Neurosurgical training simulator for cerebrovascular bypass surgery [6]	Commercial composite physical model	Vascular anastomosis techniques, tumor models also possible when applying minor modifications	Cheap, reusable, free of infection risks, extra- and intracranial circulations, haptic properties superior to other microanastomosis simulators, and radiological imaging is possible	Visual and haptic feedback inferior to animal and cadaver heads, synthetic vessels with lack of adherence to surrounding tissue
“Live cadavers “[1]	cadavers that are connected to a pump with artificial blood flow	Management of intraoperative aneurysmal rupture, clipping aneurysms, training of all procedures possible including intracranial pressure reduction, traumatic injuries, bypass, artificial brain tumors, etc.	Realistic visual and haptic feedback and blood flow, bleeding, and tissue pulsation	Time consuming preparation of the cadaver, limited availability, sanitary regulations, short shelf life with a maximum of one week, high expenses

*VR*, virtual reality; *ETV*, endoscopic third ventriculostomy; *CSF*, cerebrospinal fluid; *5-ALA*, gamma-aminolevulinic acid

In this publication, we introduce a novel simulator composed of a 3D printed skull and a calf’s brain. It was designed to improve neurosurgical training by imparting surgical and technological competencies in a well-structured, real-world scenario. The simulator needed to provide authentic haptic feedback and versatile training scenarios as well as easy and unlimited reproducibility.

## Methods

### Developmental process

In order to create a useful brain surgery training model, we focused primarily on providing realistic haptic and visual experience. The skull had to fulfill two main demands: realistic anatomical landmarks and material properties like real bone tissue particularly during trepanation, craniotomy, and drilling. A 3D printed skull consisting of plaster as a main component and displaying a lamina interna, lamina externa, diploë, and dura mater met our expectations best. After review of the respective literature and based on our own previous experience with gelatine brain models for neurosurgical training, we decided to use real brain tissue to fill the skull. Animals that show similar brain sizes as humans include dolphins or primates [17]. Obviously, availability of those brains is limited. Cow brains are not licensed for use in cadaver training in Germany due to the risk of prion contamination (regulations in other countries might differ). Due to their easy availability and low cost, we decided to use calf’s brain (similarly easily available pig brains are too small). In terms of brain tumor models, the decisive factor was that the artificial intrinsic brain tumors should be easy to obtain, biologically safe, and have similar color and haptic properties as real brain tissue. In addition, the artificial tumor should be injectable without significant damage to brain tissue, should be able to form both solid tumors and tumors with diffuse borders to simulate infiltrative growth, and should allow staining with fluorescent dye. A liquid substance that solidifies when cooled to room temperature seemed most promising, and tests showed that a mixture of aspic and water was best suited for this purpose. In order to fill the space between the calf’s brain and the skull, a substance was needed that would effectively prevent the shifting of the brain while being soft enough not to damage the brain tissue. Again, an aspic-water mixture was used, which was thicker and therefore stiffer than that used for the tumor models. It guaranteed a secure fixation of the calf’s brain and showed an advantageous homogeneous magnetic resonance imaging (MRI) signal. For fluorescence-guided surgery, a concentration of 0.0005% sodium fluorescein was identified as ideal for staining the artificial brain tumor without affecting the surrounding brain tissue or the injection site and to allow effective fluorescence guidance using the YELLOW 560 nm option of the surgical microscope (KINEVO 900, Zeiss, Oberkochen, Germany). Optionally, gadolinium (1% 0.5 mmol/L) can be added to the tumor model to facilitate tumor identification in T1-weighted MRI scans (the tumor model is clearly visible in T2).

### Simulator assembly

**Fig. 1:**
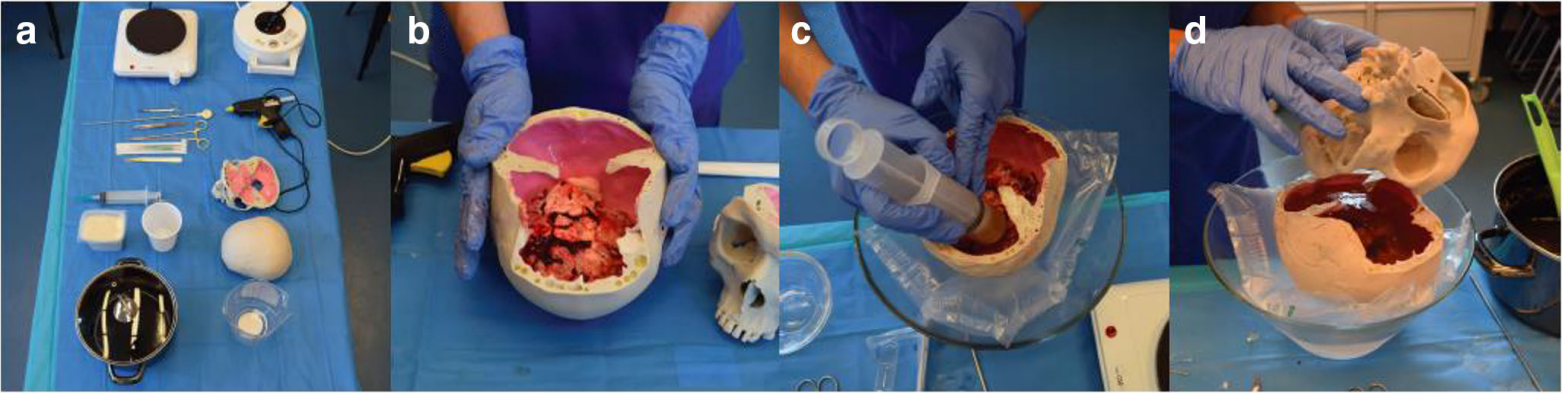
Series of pictures that show the main stages of the simulator assembly. **a** The work space for the simulator assembly. **b** The calf’s brain is placed against the convexity of the skull cap. **c** Injection of tumor masses in the frontal and occipital lobes from inferior. **d** The skull base is filled up with an aspic water solution. Then, the inverted skull cap is placed on the skull base and both parts are glued together using hot glue

A freshly extracted and cooled calf’s brain, 400 g aspic powder (beef gelatin), and a 3D printed skull consisting of separate skull base and skullcap were provided (Model Graf, PHACON GmbH, Leipzig, Germany). Both parts of the skull were made based on a human CT brain scan and comprised an artificial dura mater. For ensuring a smooth assembly, we provided the following materials: disinfectant, gloves, underpacking, calf’s brain, 3D printed skull base and skullcap, aspic powder, water, kitchen scale, measuring jug, pot, hot plate, cooking spoon, ladle, 250 ml container, 10% fluorescein, 10 μl pipette, pipette tips, 50 ml catheter tip syringe and/or 10 ml 11 gauge syringe, hemostatic forceps, disposable scalpel, sharps container, optionally 0.5 mmol/l gadolinium, and 2 ml syringe. After preparation of the work space (Fig. 1a), we boiled 1.5 l of water and slowly stirred in 200 g of aspic until complete dissolution of the aspic powder. For the tumor mass, we took 200 ml of this aspic water suspension and poured it in a separate container. Using a 10 μl pipette and a 2 ml syringe, we added 10 μl of 10% fluorescein as well as 2 ml of 0.5 mmol/l gadolinium to 200 ml of dissolved aspic and mixed it gently. We then filled a 50-ml catheter tip syringe with the tumor mixture and let it sit in cold water till it turned viscous. Alternatively, we filled a 10 ml 11 gauge syringe with the tumor mixture diluted by 200 ml water for an infiltrative tumor. In the meantime, the remaining 200 g aspic powder was added to the remaining 1.3 l aspic water solution. The resulting filling mixture slowly cooled down at room temperature for later usage. After placing the calf’s brain against the convexity of the calotte (Fig.1b), we injected 3–5 ml tumor masses in the frontal and parietal lobes from inferior (Fig. 1c). For biopsy targets, we used 1 ml tumor masses and injected them in the caudal hemispheres, midbrain or brainstem. If the tumor mixture already solidified, it could be warmed up again in hot water without removing it from the catheter tip syringe. Finally, the calf’s brain was fixed in its position using the aspic filling mass. The skull base was placed on the inverted skullcap (Fig. 1d) and both parts were glued together using hot glue. The cavity surrounding the calf’s brain was then filled up with the prepared aspic solution through the foramen magnum. Finally, the model was cooled in the freezer until the filling mass was solidified. The assembly requires approximately 1.5 h. The simulator can be stored up to 72 h in a fridge without compromising its properties.

### Surgery

**Fig. 2:**
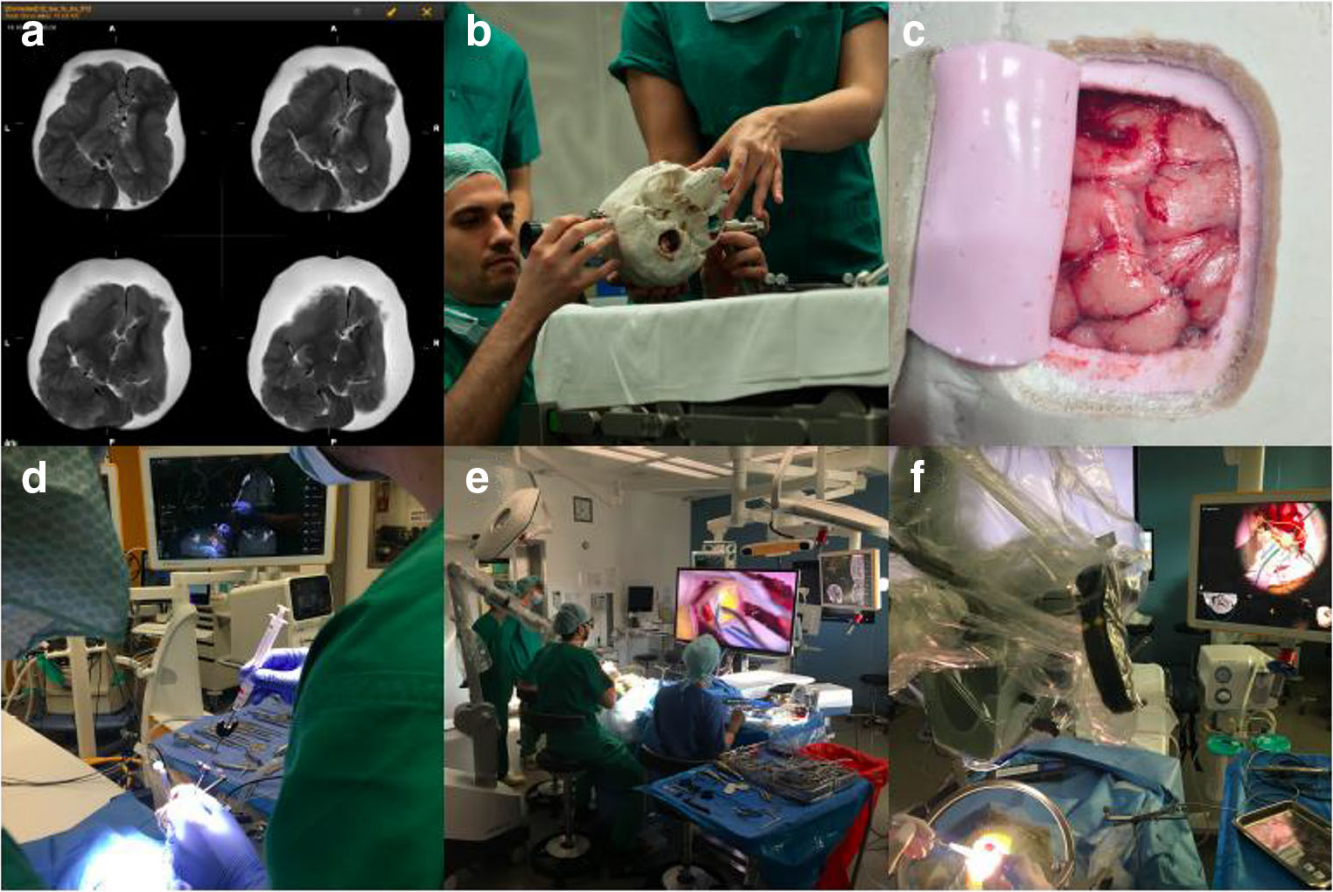
Series of pictures that show the simulator use in the operating room. **a** MRI scan of the simulator head (T2 weighted). **b** Positioning of the simulator head in the Mayfield clamp. **c** View of the simulators brain after craniotomy and dura opening. **d** Performance of a neuronavigation-guided stereotactic biopsy. **e** Performance of a brain tumor resection using fluoroscopy. **f** Performance of an augmented reality assisted brain tumor surgery.

Training scenarios were performed in the operating rooms (OR) of the Charité Department of Neurosurgery. Microscope, exoscope, neuronavigation, cavitron ultrasonic surgical aspirator (CUSA), bipolar coagulation, sucker, surgical instruments, and disposable materials were provided and set up by an experienced scrub nurse in cooperation with the neurosurgical consultant to ensure maximum workspace efficiency. Each training scenario was supported by a scrub nurse and an assistant as well as supervised by a senior consultant neurosurgeon. All team members were preferably scrubbed and paid meticulous attention to create an overall realistic impression. Training scenario performances and OR setup are exemplarily illustrated in Fig. 2.

#### Overarching learning objectives

- Software-assisted planning for a variety of intracranial procedures
- Setup and use of intraoperative neuronavigation
- Handling of surgical microscope, both ocular-based and exoscopic
- Learning robot-assisted microscope guidance using foot control
- Setup and utilization of augmented reality
- Planning of positioning and craniotomy
- Performing standard craniotomies and dura openings
- Microsurgical preparation and subpial dissection
- Use of CUSA

#### Surgical training scenarios

1. Simple tumor resection

a. Task: resection of a solid mass via frontal craniotomy

b. Learning objectives: planning anatomical and navigation-assisted surgical access, head positioning and clamping, use of exoscope, craniotomy, durotomy, dissection with bipolar and sucker, identification and removal of pathology, dural suture, and bone flap refixation

c. Target group: 1st to 3rd year of training

2. Complex tumor resection

a. Task: navigated resection of an infiltrative pathology via frontoparietal craniotomy

b. Learning objectives: planning surgical access and navigation, head positioning and clamping, use of exoscope including robotics, use of augmented reality overlay, craniotomy, durotomy, subpial dissection using CUSA, identification and removal of pathology supported by fluoroscopy, dural suture, bone flap refixation, and optionally intraoperative MRI

c. Target group: 3rd to 6th year of training

3. Navigated biopsy via burr hole trepanation

a. Task: navigated biopsy

b. Learning objectives: planning navigated biopsy, head positioning and clamping, use of navigation, burr hole trepanation, and performing a navigated biopsy

c. Target group: open

4. Retrosigmoidal craniotomy

a. Task: performing retrosigmoidal craniotomy

b. Learning objectives: planning surgical access and identification of local anatomical landmarks, head positioning and clamping, craniotomy, and closure of craniotomy

c. Target group: 3rd to 6th year of training

In the retrosigmoidal training scenario, a tumor mass in the cerebellopontine angle can be added. For this, a calf’s brain cerebellum and brainstem are placed in the cerebellar fossa and held in place with a forceps through the foramen magnum until the filling mass is solidified. This extension of the training scenario allows for preparation of the cerebellopontine angle and was successfully tested. However, we did not perform a prospective assessment here.

### Simulator costs

The simulator costs amount to approximately 575 € without gadolinium, respectively 788 € using gadolinium. They include the skull model (550 €), the calf’s brain (approximately 10 €), 400 g aspic (approximately 5 €), fluorescein (approximately 10 € per 5 ml ampulla), and optionally gadolinium (approximately 213 € per 15 ml ampulla).

### Sanitary regulations in the OR

Approval for simulator usage in operating theaters and intraoperative MRI was obtained from the local bodies responsible for patient safety. After every training, the operating theater was cleaned based on a protocol for surgical procedures in infectious patients.

### Demographics

In this study, 29 subjects participated during four workshops. Training level included consultants (*n* = 3), senior residents with 4–6 years experience (*n* = 4), junior residents with 0–3 years experience (*n* = 10), medical students (*n* = 8), no medical background (*n* = 2), and unknown experience level (*n* = 2). Experience level in craniotomies included experienced defined as 11–40 craniotomies per month (*n* = 4), little experience defined as up to 10 craniotomies per month (*n* = 9), no experience (*n* = 10), and unknown experience (*n* = 6). Experience level in tumor resections included experienced defined as more than 10 tumor resections per month (*n* = 2), little experience defined as up to 10 tumor resections per month (*n* = 8), no experience (*n* = 15), and unknown experience (*n* = 4). Age- or gender-related data were not collected. Among 27 subjects, 30% already participated in other neurosurgical simulation trainings. Two subjects gave no information here. Demographic data are given in Table 2.

**Table 2:** Demographic parameters of study participants and performed training scenarios

Training level	Total number of participants	29
	> 6 years (consultants)	3
	4–6 years (senior residents)	4
	1–3 years (junior residents)	10
	Medical students	8
	No medical background	2
	Unknown	2
Experience in craniotomies	Experienced (more than 10 craniotomies per month)	4
	Little experience (up to 10 craniotomies per month)	9
	No experience	10
	Unknown	6
Experience in tumor resections	Experienced (more than 10 tumor resections per month)	2
	Little experience (up to 10 tumor resections per month)	8
	No experience	15
	Unknown	4
Previous neurosurgical simulator trainings	Yes	8
	No	19
	Unknown	2
Number of performed training scenarios	Total number of performances	63
	Simple tumor resection	15
	Complex tumor resection	15
	Navigated biopsy	27
	Retrosigmoidal craniotomy	6

### Assessment score

Based on the rating scales Objective Structured Assessment of Technical Skills (OSATS) [14] and Northwestern Objective Microanastomosis Assessment Tool (NOMAT) [3], we created a 5-point Likert-like questionnaire (1 = the best/fully agreed/yes, 5 = the worst/completely disagreed/no) that assessed the realism of the tumor model, skull, and brain tissue as well as the capability for training purposes (Fig. 1 in the supplements).

### Statistical analysis

Descriptive statistical analysis was performed using IBM SPSS (www.ibm.com, version 24) and included calculation of median and quartiles. Results are presented as follows: median, first quartile, and third quartile. Interference and correlation analyses were not performed due to the design of the study.

## Results

### Realism of the tumor model, skull, and brain tissue

**Fig. 3:**
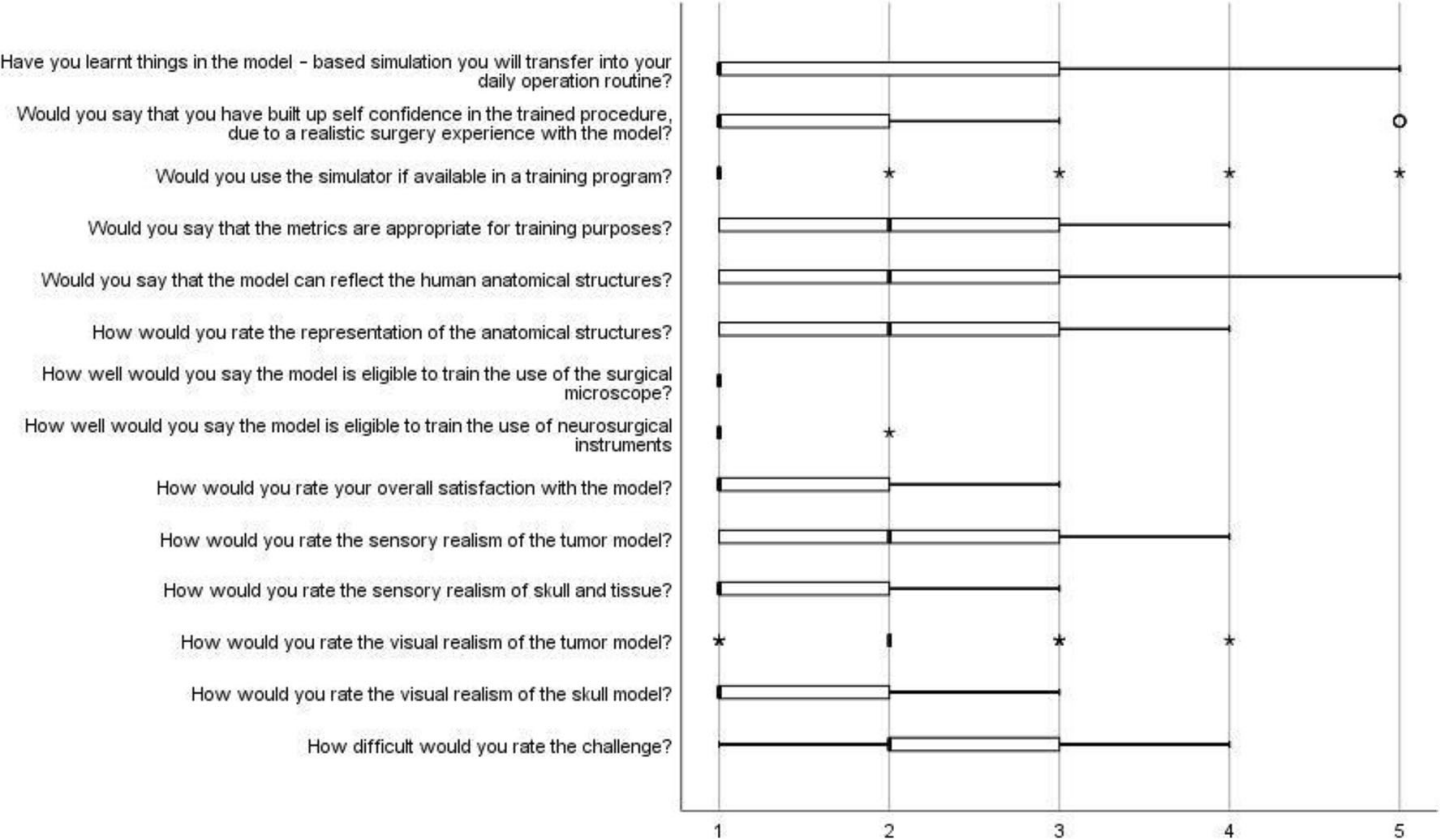
Evaluation of the 5-point Likert-like questionnaire

The sensory realism of the tumor model (2, 0; 1, 0; 3, 0) was rated “good,” while the sensory realism of the skull and brain tissue (1, 0; 1, 0; 2, 0) was rated “very good.” Likewise, the visual appearance of the tumor was “good” (2, 0; 2, 0; 2, 0) in contrast to a “very good” visual appearance of the skull (1, 0; 1, 0; 2, 0). The simulator provided a “good” representation of human anatomical structures (2, 0; 1, 0; 3, 0) and the overall satisfaction with the simulator was graded as “very good” (1, 0; 1, 0; 2, 0). Results are represented in Fig. 3.

### Capability for training purposes

Nearly all participants (90%) would use the simulator if it was available in their training program (1, 0;1, 0;1, 0). The anatomical features of the model for training purposes were rated “very good” (1, 0;1, 0;1, 0). Likewise, the eligibility of the surgical microscope (1, 0;1, 0;1, 0) and neurosurgical instruments (1, 0;1, 0;1, 0) for training purposes were rated “very good.” The difficulty of the challenge was rated as medium (2, 0;2, 0;3, 0). The majority of participants stated that they learnt things during the simulation training, which they will transfer into their daily routine (1, 0;1, 0; 3, 0) and that they grew in confidence in the trained procedure due to realistic surgery experience (1, 0;1, 0;2, 0).

### Qualitative interviews

The setup of the neuronavigation was assessed as “challenging” if only T1-weighted magnetization prepared rapid gradient echo (MPRAGE) sequence was available for registration of the simulator head. However, subsequent fusion with a CT scan provided by the manufacturer of the skull revealed a distinct improvement of the neuronavigation workflow and accuracy. Moreover, participants reported difficulties in distinguishing brain tissue from tumor masses in T1, resembling non-enhancing gliomas. Based on this feedback, we generated a tumor mass that contained gadolinium contrast agent. This approach was well received, especially by younger residents. Alternatively, T2-weighted images enabled a better differentiation of tumor masses and surrounding brain tissue. In addition, participants described the possibility to practice surgical scenarios on real brain tissue and use of latest technology in a real-life scenario as an exceptionally good experience—including those with previous surgical training experience. However, the small size of calf’s brains was negatively acknowledged and resulted in a limited variety of surgical scenarios. Also, the lack of perfusion and the inability to simulate bleedings was identified as a definite shortcoming.

## Discussion

Neurosurgery is characterized by challenging procedures as well as rapidly developing technical devices and methods. In surgery, 75% of errors are preventable and technical in nature [7]. A recent study claimed that neurosurgical residency in Europe lacks training concepts that address the issue of complex surgical procedures and use of technology [18]. Simulator models based on artificial tissue, VR environments, and cadavers have been proposed to meet this challenge. However, these models lack realistic haptic feedback, visual feedback, and/or simulator availability [7]. Also, training efficiency typically suffers from low emotional involvement due to unrealistic scenarios. Artificial brain tissue from 3D printing or cast model approaches has so far not offered sufficient haptic realism compared with real brain tissue.. Even high-end composite materials struggle in terms of elasticity, overall haptic properties, and visual realism. Trainees usually recognize simulators as being artificial with the result of a potential decrease in training efficiency and long-lasting learning experience, since these are related to emotional experience during the training process. Safety improvement using simulations necessitates full integration of its applications into structures and practices of routine health care [9]. Another approach for surgical training is VR. It allows not only practicing simple surgical procedures, but even complex scenarios and microsurgical techniques can be performed [15, 19]. However, haptic feedback solutions are still very simple [13]. Beyond which, it is currently not possible to properly simulate the presurgical planning and intrasurgical technological support in VR. Nevertheless, VR technology is a promising tool, which is already useful for several training purposes as the increasing number of publications illustrate (Table 1). Human cadavers on the other hand exhibit great visual and haptic realism, but their significant drawback is a limited availability and strict regulations in terms of place and purpose of their usage. As a result, access to cadaver trainings is rare and typically involves high expenses. Also, the use of human cadavers for basic training purposes can be regarded as ethically controversial and not contemporary.

### Strengths

In this study, we introduced a novel simulator that combines artificial and real tissue to overcome the issues of unrealistic haptic feedback and limited simulator availability. The novel training model offers training of various procedures including craniotomy, biopsy, tumor resection, handling of technical devices, and software. The simulator training improves competencies of surgery-related tasks like review of MRI scans, determination of the proper surgical approach, head positioning, sterile draping, usage of microscope and fluorescein, and identification of neuroanatomical landmarks. The materials are easily accessible and provide detailed anatomical structures. The model is very suitable for practicing skills necessary to perform safe brain tumor surgery with the exception of bleeding control. As stated by all study participants, another strength of this model is the capability to perform a complete surgical procedure, including state-of-the-art technological support with very good sensory and visual feedback of the skull and brain, and the opportunity for microsurgical resection of a fluorescent tumor model with good sensory and visual realism. Previous studies have shown that realistic haptic feedback is crucial for effective surgical training. Better haptic feedback results in an improved skill development of the trainee compared with training with limited haptic feedback [2, 4]. The assembly of the simulator requires approximately 90 min and can easily be performed by people without a medical background.

### Weaknesses

Although the representation of the anatomical structures was rated as good, the model does not reflect the human anatomy completely satisfactorily due to the small size of the calf brain compared with the human brain. In addition, invariable factors such as the quality of the calf brain extraction, the time interval between calf brain extraction and purchase, or the room temperature during training can influence the quality of the simulator. However, this barely affected the authenticity of the training model and had no effect on the representation of anatomical structures. Major drawbacks of our model are its limited shelf life due to the usage of a real brain, the amount of time for model assembly, and no training of bleeding control due to absence of blood flow and tissue pulsation. Although the high expenses of approximately 600 € represent a downside of the simulator, they will expectably decrease when 3D printing technology will become more broadly available and cheaper.

### Impact on neurosurgical skills and prospects

The observational and self-reporting study design did not allow for conclusions about the model’s objective effect on skill development. A prospective analysis of the correlation between the difficulty of the scenarios and the learning effect is a necessary next step to validate the model. However, it has already been observed that the more difficult the participants assessed the trained procedure, the greater the increase in confidence. It can therefore be assumed that the learning objectives of more difficult procedures are more likely to be transferred into the daily routine. However, our data were not sufficient for a statistical analysis of this phenomenon. In order to further explore this aspect, regular workshops with participants at different levels of training need to be held. In addition, the performance of the participants in the operating theater before and after the training must be evaluated in order to examine the effects of the training. In addition to qualitative interviews and questionnaires to record subjective learning success and increase in self-confidence, objective checklists for benchmarking of individual skills must be established.

In addition, for the goal of simulation-supported learning during the entire residency, further neurosurgical procedures that are not limited to brain tumor resections must be covered. Aboud et al. presented a scenario in which cadavers are equipped with a pump to create an artificial blood flow. In theory, such a pump could also be installed in our model, but it would require the calf’s brain to be delivered immediately after extraction from the calf and would significantly complicate the assembly process. As a result, however, vascular procedures and bleeding control in tumor resections could be trained. Flexible training scenarios could also be created by creating different variants of the 3D printed skull. For example, 3D skulls including an artificial dura mater could be designed to simulate traumatic brain injuries, pediatric tumor resections, meningiomas, or epidural bleeding. Further refinement of the 3D printing process should, among others, also enable scenarios for transsphenoidal resection of pituitary adenomas.

In conclusion, this novel training model provides a powerful alternative to artificial-, VR-, or cadaver-based simulators and allows for several brain tumor scenarios with high training efficiency. Since this model is composed of a 3D printed skull and a calf’s brain, it is hallmarked by very good haptic and visual authenticity as well as unlimited reproducibility. The drawbacks are small size of the calf’s brain, short shelf life, duration of model assembly, pricing, no training of bleeding control, and absence of blood flow and tissue pulsation. Based on the scenarios of this study, especially early career neurosurgeons benefit from the novel simulation setup.

## Electronic supplementary material

ESM 1 (DOCX 18 kb)
