# Supplementary material for: Development and initial evaluation of a novel simulation model for comprehensive brain tumor surgery training
Source: Acta Neurochir (Wien). 2020 May 8;162(8):1957–65. doi: 10.1007/s00701-020-04359-w (PMC7360639; doi:10.1007/s00701-020-04359-w)
Supplement: Supplementary file 1 — (DOCX 18 kb) [file 701_2020_4359_MOESM1_ESM.docx]

## Are you a neurosurgical resident?

- Yes
- No

## If yes, how advanced are you with your residency?

- First year
- Second year
- Third year
- Fourth year
- Fifth year
- Sixth year
- > Sixth year

## How many own tumor resection surgeries have you performed in average per month over the last three months (best estimate)?

- None
- 1-3
- 4-10
- 11-20
- 20-40
- >40

How many own craniotomies on a patient have you performed in average per month over the last three months (best estimate)?

- None
- 1-3
- 4-10
- 11-20
- 20-40
- >40

## Have you done neurosurgical simulation training before?

- Yes
- No

## How do you generally feel about your skills in the trained operation technique?

- I’m generally feeling very good about my overall performance in the operation theatre for the trained technique
- I’m generally feeling very good about my performance as long as an experienced doctor is present in the operation theatre or is in direct reach for me
- I’m generally not sure about the trained technique due to my lack of experience in the trained methods
- I have never performed the trained operation before

Rate the following questions from 1 to 5, with one being “the best/fully agreed/yes” and five being “the worst/completely disagreed/no”.

|  | 1 | 2 | 3 | 4 | 5 |
| --- | --- | --- | --- | --- | --- |
| How difficult would you rate the challenge? |  |  |  |  |  |
| How would you rate the visual realism of the skull model |  |  |  |  |  |
| How would you rate the visual realism of the tumor model |  |  |  |  |  |
| How would you rate the sensory realism of skull and tissue |  |  |  |  |  |
| How would you rate the sensory realism of the tumor model |  |  |  |  |  |
| How would you rate your overall satisfaction with the model |  |  |  |  |  |
| How well would you say the model is eligible to train the use of neurosurgical instruments |  |  |  |  |  |
| How well would you say the model is eligible to train the use of the surgical microscope |  |  |  |  |  |
| How would you rate the representation of the anatomical structures |  |  |  |  |  |
| Would you say that the model can reflect the human anatomical structures |  |  |  |  |  |
| Would you say that the metrics are appropriate for training purposes |  |  |  |  |  |
| Would you use the simulator if available in a training program? |  |  |  |  |  |
| Would you say that you have built up self confidence in the trained procedure, due to a realistic surgery experience with the model? |  |  |  |  |  |
| Have you learnt things in the model – based simulation you will transfer into your daily operation routine? |  |  |  |  |  |

…Thank you very much for your participation!
